# EXamining ouTcomEs in chroNic Disease in the 45 and Up Study (the EXTEND45 Study): Protocol for an Australian Linked Cohort Study

**DOI:** 10.2196/15646

**Published:** 2020-04-14

**Authors:** Celine Foote, Carinna Hockham, Louisa Sukkar, Anna Campain, Amy Kang, Tamara Young, Alan Cass, Clara K Chow, Elizabeth Comino, Martin Gallagher, Stephen Jan, John Knight, Bette Liu, Martin McNamara, David Peiris, Carol Pollock, David Sullivan, Germaine Wong, Sophia Zoungas, Kris Rogers, Min Jun, Meg Jardine

**Affiliations:** 1 The George Institute for Global Health Sydney Australia; 2 Concord Repatriation General Hospital Sydney Australia; 3 Faculty of Medicine University of New South Wales Sydney Australia; 4 School of Public Health University of Sydney Sydney Australia; 5 Menzies School of Health Research Charles Darwin University Darwin Australia; 6 Westmead Applied Research Centre Faculty of Medicine and Health University of Sydney Sydney Australia; 7 Department of Cardiology Westmead Hospital Sydney Australia; 8 Centre for Primary Health Care and Equity University of New South Wales Sydney Australia; 9 Sydney Medical School University of Sydney Sydney Australia; 10 School of Public Health and Community Medicine University of New South Wales Sydney Australia; 11 The Sax Institute Sydney Australia; 12 Renal Division Kolling Institute for Medical Research Sydney Australia; 13 University of Sydney Sydney Australia; 14 Department of Chemical Pathology Royal Prince Alfred Hospital Sydney Australia; 15 Centre for Transplant and Renal Research Westmead Hospital Sydney Australia; 16 School of Public Health and Preventive Medicine Monash University Melbourne Australia; 17 Graduate School of Health University of Technology Sydney Sydney Australia

**Keywords:** chronic kidney disease, diabetes mellitus, cardiovascular disease, data linkage, biomarkers

## Abstract

**Background:**

Chronic kidney disease (CKD) and diabetes are the major causes of death and disability worldwide. They are associated with high health service utilization persisting over many years. Their slow progression and wide clinical variation make them eminently suitable for study in population-based cohorts. However, current understanding of their prevalence, incidence, and progression is largely based on studies conducted in clinical populations.

**Objective:**

This study aims to establish a novel link between an existing population-based cohort (the 45 and Up Study) and routinely collected laboratory and administrative data to facilitate research across the full disease spectrum of CKD and diabetes.

**Methods:**

In the EXTEND45 Study (EXamining OuTcomEs in chroNic Disease in the 45 and Up Study), baseline questionnaire responses of over 260,000 participants of the 45 and Up Study aged ≥45 years living in New South Wales (NSW), collected between January 2006 and December 2009, are linked to data from laboratory service providers as well as national- and state-based administrative datasets via probabilistic linkage. Routinely collected data were obtained for participants who could be linked between January 2005 and July 2013. Laboratory data will enable the identification of early cases of chronic disease and the assessment of clinically relevant biochemical targets during the disease course. Health administrative datasets will allow for the examination of health service use, pharmacological management, and clinical outcomes.

**Results:**

The study received ethics approval from the NSW Population and Health Services Research Ethics Committee in February 2014. Data linkage for 267,153 of the 45 and Up Study participants was completed in June 2016, with congruent linkage achieved for 265,086 (99.23%) individuals. To date, the CKD and diabetes cohorts have been identified (published elsewhere), and a diverse portfolio of research projects relating to disease burden, risk factors, health outcomes, and health service utilization is in development.

**Conclusions:**

The EXTEND45 Study represents an unparalleled opportunity to perform extensive research into diseases of considerable public health and clinical importance. Strengths include the population-based nature of the cohort and the availability of longitudinal information on the complete disease pathway for affected individuals.

**International Registered Report Identifier (IRRID):**

RR1-10.2196/15646

## Introduction

Chronic diseases such as chronic kidney disease (CKD) and diabetes are major drivers of death [1], disability [2], and health care costs globally [3-5]. In Australia, 1.7 million people had indicators of CKD based on biomedical testing in 2011-2012 [6], whereas recent estimates for diabetes suggest that 1.2 million individuals were living with type 1 or 2 diabetes in 2017-2018 [7]. Adults aged 65 years and older bear the majority of this burden. Estimates vary according to study context and design, but most studies indicate at least a twofold increase in the prevalence of diabetes or CKD in people aged >65 years compared with their younger counterparts [8-11]. As the population ages, this problem is expected to further increase [12], with important consequences for already stretched health systems.

Diabetes and CKD are intrinsically linked [13,14], with diabetes being an important risk factor for CKD, and both independently associated with an increased risk of cardiovascular disease (CVD) [15]. The relative risk of CVD in adults with diabetes compared with those without ranges from 1 to 3 in men and 2 to 5 in women [16]. Similarly, individuals with impaired kidney function and increased urinary albumin excretion have a twofold to fourfold higher risk of developing CVD than those whose kidney function is normal [17]. In patients with established CVD, coexisting diabetes or CKD is associated with worse outcomes and a higher mortality rate [18-20]. Early detection and timely intervention are, therefore, paramount to the prevention and management of this trifecta of conditions.

Although there is a growing understanding of the prevalence and progression of diabetes, CKD, and their CVD-related complications, many studies to date have taken place in clinical populations recruited through specialist clinics and hospital settings. Evidence for effective management strategies, including clinical targets (eg, for glycated hemoglobin [HbA_1c_] in diabetes) and different therapy options (eg, renin-angiotensin-aldosterone blockade in CKD), largely stems from randomized trials conducted in controlled clinical settings with narrowly selected patient populations. However, the slowly progressive nature of diabetes and CKD, coupled with their wide clinical variation, means that earlier or milder stages of disease are missed in hospital- or clinic-based cohorts. More population-based research into these diseases is needed to (1) gain detailed knowledge of the incidence and prevalence of diabetes and CKD in the community, including earlier disease stages, (2) identify novel determinants of disease progression, (3) better assess the broad-scale benefits and adverse effects of evidence-based therapies, and (4) evaluate health care utilization, costs, and outcomes across the full disease spectrum.

Direct assessment of the various facets of chronic disease burden and management in prospective cohorts is a major undertaking. To generate reliable, generalizable estimates, cohort studies must include a sufficiently large sample to capture most cases and be adequately distributed, both geographically and socioeconomically. In addition, rigorous follow-up of study participants is necessary, which is costly and often results in high loss to follow up. Routinely collected and administrative clinical data—collected as a by-product of patient care—offer an alternative method for acquiring detailed longitudinal information on health service use, disease burden, and clinical outcomes in a large proportion of the population.

The 45 and Up Study is a large-scale study established in 2006 that combines the merits of a prospective population-based cohort study and linked administrative health data [21]. Comprising more than 260,000 individuals aged ≥45 years living in New South Wales (NSW), the 45 and Up Study was designed to obtain information on healthy aging through questionnaires delivered to participants. In addition, participants consented to their questionnaire responses being linked to routinely collected health data for research.

The infrastructure generated through the 45 and Up Study offers the unique opportunity to examine diabetes, CKD, and their associated health outcomes in the community. However, to date, there has been no effort to utilize valuable data generated from routine laboratory testing to examine early disease states or assess the achievement of clinical targets in patients with CKD, diabetes, or both. The EXTEND45 Study (EXamining ouTcomEs in chroNic Disease in the 45 and Up Study) builds upon the 45 and Up Study by establishing a novel link between the 45 and Up Study participants and their clinical data held by laboratory service providers. The EXTEND45 Study will concurrently link the 45 and Up Study participants to a range of other administrative datasets to obtain information across the full spectrum of disease and health service provision, spanning 10 years.

The mission of the EXTEND45 Study is to establish a rich data resource to examine CKD, diabetes, and their CVD-related complications. Initial research objectives of the EXTEND45 Study include the following:

1. To determine the prevalence and incidence of CKD in the 45 and Up Study cohort and define a seminal CKD cohort that can be used in future studies;
2. To determine the prevalence and incidence of diabetes in the 45 and Up Study cohort and define a seminal diabetes cohort that can be used in future studies;
3. To determine the prevalence and incidence of CKD in individuals identified as having diabetes in the 45 and Up Study cohort;
4. To identify risk factors for CKD and diabetes in the community;
5. To examine the real-world management of these diseases, including prescribing patterns, and identify evidence-practice gaps in the care of individuals with CKD, diabetes, or both, particularly in relation to CVD prevention; and
6. To evaluate the attainment of clinical targets in the community, identify risk factors for nonattainment, and assess associated health outcomes.

This paper provides a detailed description of the data sources, linkage methods, and governance structure of the EXTEND45 Study.

## Methods

### The Australian Health System

Australia has a universal health system comprising a multifaceted network of government (public) and private providers. Medicare is the universal public health insurance scheme, which is funded by the federal government through a combination of general tax revenue and a Medicare levy based on taxable income. It is administered by Services Australia (formerly the Department of Human Services) and provides free or subsidized access to 3 main areas of health care provision: (1) medical and health services outside of the public hospital setting (administered through the Medicare Benefits Schedule [MBS]), (2) prescription pharmaceuticals (administered through the Pharmaceutical Benefits Scheme [PBS]), and (3) public hospital services (funded jointly with, and managed by, state and territory governments). In addition, the federal government pays 75% of the fee for services and procedures for private patients in public or private hospitals and subsidizes private health insurance. The EXTEND45 Study includes administrative data collected by the federal government (ie, MBS and PBS) as well as state-specific data collections of the NSW government.

### The 45 and Up Study

Details of the 45 and Up Study have been published previously [21]. Briefly, the 45 and Up Study comprises adults aged ≥45 years sampled from the general population of NSW, Australia’s most populous state. Between January 2006 and December 2009, participants were randomly sampled from the Services Australia enrollment database, with individuals aged ≥80 years or living in remote areas oversampled by a factor of 2. Consenting participants self-completed baseline questionnaires in English, which included questions on demographic and socioeconomic characteristics, personal health behaviors, medical and surgical history, medications, and physical and psychological health [22]. Participants also consented to long-term follow-up, including linkage of their baseline responses to routinely collected health datasets. In the EXTEND45 Study, laboratory and administrative datasets have been linked at an individual level to the 45 and Up Study participants and their baseline questionnaire responses.

### Linked Data Sources

#### Laboratory Service Providers

**Figure 1:**
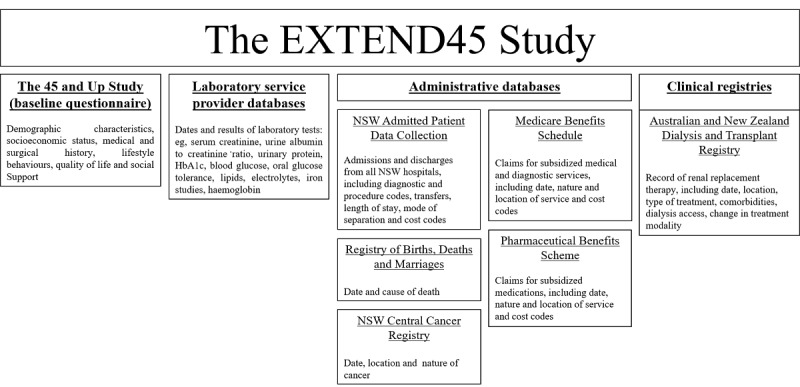
An overview of the EXTEND45 Study (EXamining OuTcomEs in chroNic Disease in the 45 and Up Study) data sources and variables. NSW: New South Wales.

Government datasets hold information on referrals for community laboratory services but not the clinical results of those services. Instead, these are held by the laboratory service providers who conduct the tests, together with patient identifiers, tests performed, and date of testing (Figure 1). Multiple public and private laboratory service providers are active within NSW, and these vary in geographical scope and population coverage. In addition, providers offer varying levels of access to community, outpatient, or inpatient laboratory services, thereby capturing health information across different facets of an individual’s health journey. Several major community laboratory service providers have been recruited for the EXTEND45 Study, and processes are underway to include data from an inpatient provider.

Available laboratory results and their meta-data between January 2005 and October 2015 are included at present. These enable early case identification (eg, serum creatinine measurements for kidney disease) as well as the assessment of disease progression, presence of disease complications (eg, albuminuria in patients with diabetes), and achievement of therapeutic targets (eg, HbA_1C_ in patients with diabetes and hemoglobin levels in patients with CKD).

#### New South Wales Admitted Patient Data Collection

The NSW Admitted Patient Data Collection (NSW APDC) collates information on all admitted patient services provided by public (including psychiatric) and private hospitals, private day procedures centers, and public multipurpose services in NSW [23]. For public hospitals, data are recorded for each episode of care, with each episode defined as a period of stay in hospital before the patient is discharged or transferred, dies, or becomes a different *type* of patient (eg, acute, palliative, and rehabilitative). For private hospitals, each APDC record represents a complete hospital stay. Data include dates of admission and separation, referral source, diagnoses (including external causes), procedures, and service referred to on discharge. Up to 50 relevant diagnoses are included and are coded according to standardized codes from the International Statistical Classification of Diseases and Related Health Problems, Tenth Revision, Australian Modification (ICD-10-AM).

At present, the EXTEND45 Study includes APDC data on separations that occurred between January 2005 and June 2014. NSW APDC data will be used to assess health service utilization, length of stay, referral and transfer patterns, and costs of hospital care (Figure 1).

#### Medicare Benefits Schedule and Pharmaceutical Benefits Scheme

Medicare operates by paying a specified benefit (in the form of a rebate) for services or prescription medicines that qualify for a benefit under the MBS and PBS, respectively, and for which a claim has been processed. As such, the MBS data collection contains information on all claims for medical and diagnostic services (including laboratory testing) provided to Australian citizens and permanent residents by registered medical and other practitioners, whereas the PBS data collection records all claims for prescription medicines above the PBS copayment threshold. From April 2012, suppliers of PBS medicines were required to provide data on the dispensing of medicines below the copayment threshold. MBS services and PBS items are coded using a system of item numbers listed in the relevant schedules.

MBS and PBS data are available from June 2004 to December 2016. MBS data will be used to assess health service utilization in primary care and outpatient settings, costs of care, and referral patterns. PBS data will be used to examine medication adherence, persistence, and cost, as well as the geographical distribution of services.

#### New South Wales Registry of Births, Deaths, and Marriages

All deaths are certified by a medical practitioner and registered by the NSW Registry of Births, Deaths, and Marriages (NSW RBDM). Details include cause and date of death, with cause of death coded using the ICD-10-AM coding system.

Data are available from February 2006 to March 2015. Access to NSW RBDM data will allow the comprehensive assessment of mortality, including primary and secondary causes of death.

#### Australian and New Zealand Dialysis and Transplant Registry

The Australian and New Zealand Dialysis and Transplant (ANZDATA) Registry maintains records of all patients in Australia and New Zealand with end-stage kidney disease who receive chronic renal replacement therapy (RRT), that is, dialysis or transplantation. The registry records the date of referral, start date of RRT, treatment modalities and vascular access for dialysis (both initial and any changes), and treatment outcomes for all patients.

Linkage to the ANZDATA Registry will capture information pertaining to the progression of kidney disease, including dialysis initiation or kidney transplantation. In addition, linkage of practice factors, such as treatment modalities and vascular access, to information on participant socioeconomic status and health service use may help to identify novel areas for intervention (Figure 1).

#### New South Wales Central Cancer Registry

The NSW Central Cancer Registry (NSW CCR) is a registry of all patients with cancer in NSW. The data collected relates to invasive primary cancers and cancer deaths. It does not include skin cancers other than melanoma (eg, basal cell carcinoma and squamous cell carcinoma are excluded). Data collected by the NSW CCR includes clinical details describing the cancer, records of care from a notifier, pathology reports, and death certificates.

The inclusion of cancer registry data will allow further delineation of chronic disease groups at risk of cancer complications, with respect to comorbidities, lifestyle, socioeconomic status, and health service utilization.

### Data Linkage Methods

Record linkage brings together information on the same individual from different data sources. Strict privacy protecting protocols are mandated by the NSW Population and Health Services Research Ethics Committee (PHSREC), the data custodians, and the NSW Center for Health Record Linkage (CHeReL). The CHeReL is an intermediate body established in 2006 to provide expertise in data linkage methodology and maintain a record linkage system that protects data privacy. It is jointly managed by the NSW Ministry of Health and the NSW Cancer Institute.

**Figure 2:**
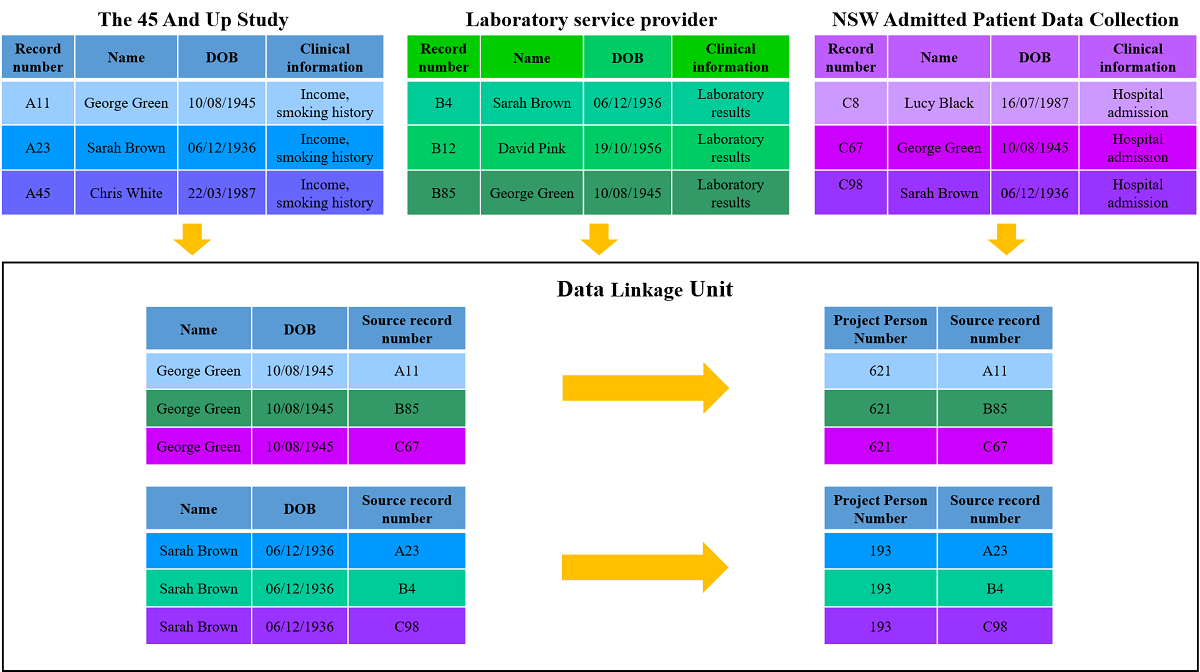
A schematic diagram of the linkage process: Stage 1. Each data custodian provides the Centre for Health Record Linkage (CHeReL, a data linkage unit) with relevant personal information of individuals who have accessed their service, together with their unique record number. The names and details used in the figure are fictitious and do not relate to any participants of the 45 and Up Study. The CHeReL uses an algorithm to match individuals to participants of the 45 and Up Study and assigns a project-specific person number to each individual. DOB: date of birth.

**Figure 3:**
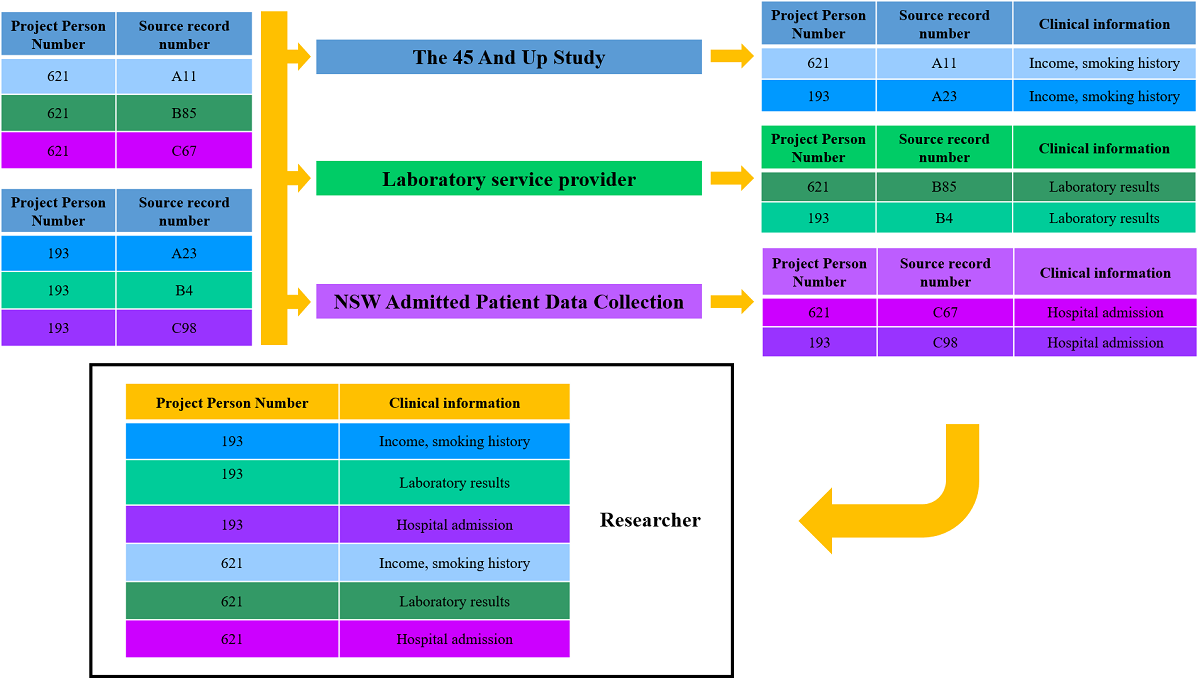
A schematic diagram of the linkage process: Stage 2. The Centre for Health Record Linkage (CHeReL) provides data custodians with the project-specific person number (PPN) for each individual, with all personal information removed. Data custodians use the PPN, together with their own record numbers, to extract the requested data and upload the data to Secure Unified Research Environment.

The Sax Institute has an ongoing link with Services Australia, and as a result has processes in place to deterministically link MBS and PBS data to their 45 and Up Study participants once a year. This is done using a unique identifier provided to the Sax Institute by Services Australia. For all other aforementioned datasets, linkage to 45 and Up Study data is performed by the CHeReL using probabilistic linkage (Figures 2 and 3). Data custodians provide the CHeReL with an encrypted unique identifier and relevant personal information (full name, date of birth, address, gender, and, where available, country of birth) for all patients over the relevant time-period. No clinical data are provided to the CHeReL at any point in the linkage process. The CHeReL uses personal information to link records from different data sources, using probabilistic linkage in the *ChoiceMaker* software (ChoiceMaker Technologies, Inc; Figure 2). For records with doubtful matches, a clerical review is also conducted [24]. Once records are linked, the corresponding individual is allocated a project-specific person number (PPN). The CHeReL sends the PPNs for all individuals to each data custodian, together with the original unique identifier used in that custodian’s particular dataset (Figure 3). Each data custodian will now hold an EXTEND45-specific PPN for each individual in their respective dataset for whom linkage with at least one other dataset was achieved. The data custodian merges the PPN with the clinical variables that have been approved for use in the EXTEND45 Study and uploads the deidentified data to a highly secure server that is accessed by the EXTEND45-approved researchers. The PPN is used to combine records for the same person from different datasets.

### Ascertainment of Disease Status

The 2 initial diseases of interest are CKD and diabetes. For each individual, their diabetes and CKD status and, where appropriate, timing of incident disease, are determined using the various linked datasets. The precise methods used to ascertain disease status will be outlined in the relevant publications. In general, however, kidney function and CKD status are predominantly derived using data from laboratory records and, where appropriate, the ANZDATA Registry. Linked serum creatinine measurements are applied to the Chronic Kidney Disease Epidemiology Collaboration equation to calculate the estimated glomerular filtration rate [25]. Other available laboratory data related to kidney function include urine albumin-to-creatinine ratio and urine protein-to-creatinine ratio.

Diabetes status is derived using the following criteria: (1) recorded dispensing of insulin (Anatomical Therapeutic Chemical [ATC] Classification: A10A) or oral blood glucose-lowering medication (ATC Classification: A10B) in the PBS dataset, (2) self-reported diabetes on the 45 and Up Study (answered *Yes* to Q24. *Has a doctor EVER told you that you have diabetes?*), (3) laboratory record of HbA_1c_ result >6.5% on one occasion, (4) laboratory record of fasting plasma glucose levels >7.0 mmol/L, and (5) laboratory record of serum glucose levels >11.1 mmol/L. At present, no distinction has been made between type 1 and type 2 diabetes.

### Study Governance

The EXTEND45 Study is led by a collaborative group of physicians, epidemiologists, and statisticians coordinated by The George Institute for Global Health, Sydney. Similar to the 45 and Up Study, the EXTEND45 Study has been designed to facilitate cooperative projects that lie within its scope, with oversight provided by a designated steering committee. The steering committee includes representatives from The George Institute for Global Health, the Universities of NSW and Sydney, the Sax Institute, clinical experts from Australian hospitals, and nonvoting representatives from pharmaceutical sponsors. Current steering committee members are listed in Multimedia Appendix 1.

The steering committee is responsible for the general oversight and governance of the study and provides academic independence and integrity. It provides scientific advice regarding all aspects of study design, conduct, analyses, and publication of results. In addition, the steering committee is responsible for the approval of prespecified analysis plans. Analysis proposals from internal or external researchers are encouraged and may be submitted by academic investigators, health service providers, or commercial health care entities.

### Ethical Considerations and Data Privacy

Data collection for the EXTEND45 Study was through data linkage only. Participants are included on the basis of their participation and consent to the 45 and Up Study, which included consent for linkage to their routinely-collected health records. The 45 and Up Study was approved by the University of NSW Human Research Ethics Committee (UNSW HREC). Ethical approval for the EXTEND45 Study was obtained from the NSW Population and Health Service Research Ethics Committee (HREC/13/CIPHS/69).

Participant confidentiality is protected in several ways. First, linkage methodology splits the processes of record linkage and data analysis, ensuring that participant identifiers are always kept separate from clinical data. Second, security measures in place at the CHeReL ensure that the risks of a breach of privacy are minimal. Third, as mandated by the Sax Institute, all data within the EXTEND45 Study is transferred and accessed via the Secure Unified Research Environment (SURE). SURE is a high-performance remote-access virtual computing environment designed specifically for secure access, storage, and analysis of anonymized health information [26]. To prevent data from being exchanged between different linked data studies, each study in the SURE facility exists within its own security perimeter.

## Results

The 45 and Up Study cohort is relatively heterogeneous, with variation observed across most variables [27]. Of the 267,153 participants of the 45 and Up Study who completed the baseline questionnaire, data linkage was performed for 266,969 (99.93%). Incongruent dates, for example, between the 45 and Up Study enrollment and recorded date of death, or other erroneous dates were identified for 1883 individuals. The final linked dataset, therefore, comprises 265,086 individuals. As of November 2019, laboratory data are available for 152,169 (57.40%) individuals, with plans for expansion ongoing. Prevalent and incident cases of CKD and diabetes have been identified from multiple data sources, and the corresponding prevalence and incidence estimates have been presented in separate publications. A diverse portfolio of research questions relating to CKD and diabetes and their management in the community are currently underway.

## Discussion

### Principal Findings

Through a novel linkage between the 45 and Up Study, various administrative databases, and data from laboratory service providers, the EXTEND45 Study represents a unique and rich data resource that can be used to investigate a range of questions relating to chronic disease. The study’s mission is to provide much-needed evidence of the epidemiology, burden, progression, and clinical management of chronic diseases such as CKD and diabetes in the general Australian population and across the full disease spectrum. Multiple projects that use this data resource are already completed or underway.

Altogether, the datasets included in the EXTEND45 Study provide a more complete overview of the care provided to patients with chronic disease than is currently available. The 45 and Up Study baseline questionnaire included questions on a range of personal health behaviors (eg, smoking, alcohol consumption, physical activity, and sleep habits) as well as demographic, social, and economic characteristics (eg, marital status, country of birth, and education level). Although many of these factors are either known or hypothesized to be important confounders of chronic disease incidence and progression, they are typically poorly captured in administrative datasets. Conversely, the 45 and Up Study baseline data provide only a single snapshot in time, are exclusively self-reported, and are lacking in granularity. In the EXTEND45 Study, the combining of NSW APDC and other health datasets will allow outcomes, comorbidities, and complications of CKD and diabetes to be examined. Moreover, laboratory data are critical for identifying individuals at the early stages of their disease and allow more granular assessment of the achievement (or lack thereof) of clinical targets. Finally, the collection of postcodes of residences and health care facilities in the various datasets will enable the geographic evaluation of access and use of all tiers of health services.

The effective use of routinely collected data and, in particular, laboratory data in chronic disease research is demonstrated by the many research and policy outputs of the ongoing Alberta Kidney Disease Network (AKDN). The AKDN is a collaborative research organization that holds a central repository of linked laboratory and administrative data from Alberta, Canada [28]. Research using this repository has ranged from a comparative risk assessment of coronary events in patients with diabetes and CKD [20] to factors associated with RRT initiation [29]. In the United Kingdom, the UK Biobank, established in 2006-2010, has recruited 500,000 people aged 40 to 69 years to provide detailed information about themselves, undergo various measurements, provide biological samples for analysis, and have their electronic health record data linked, to create a major data resource. To date, 989 papers have been published that used this resource. Similarly, the UK Clinical Practice Research Datalink (CPRD) links general practice records to secondary care, mortality, and other disease-specific databases. The CPRD is an excellent example of the utility of real-world data in generating evidence for drug safety guidance and clinical practice in particular. Research carried out as part of the EXTEND45 Study will complement these earlier studies.

### Strengths and Limitations

A main strength of the EXTEND45 Study is the breadth and diversity of the data held within it, allowing analyses into a range of research questions to be conducted. Some of the potential analyses have already been described. Importantly, the inclusion of laboratory data allows researchers to use the EXTEND45 Study dataset to identify earlier stages of both diabetes and CKD and move away from reliance upon self-report for diabetes. This will provide essential information on the prevalence and progression of early disease in the community as well as health care delivery to those affected with mild disease. Indeed, previous studies have either relied on clinical populations or used sources that only include outcome measures such as hospitalization or mortality—in both instances, chronic disease is identified later in its disease course. Second, multiple data sources will be used to ascertain chronic disease status, which will improve case identification. The relative contributions of different data sources to the identification of cases will be reported in the respective publications. Finally, the combined infrastructure of the 45 and Up Study and EXTEND45 Study offers a cheaper and more efficient alternative to traditional longitudinal cohort studies for assessing the incidence of disease, health outcomes, and health service utilization in a large cohort.

There are important limitations that must be considered when using the EXTEND45 Study database, both at the design stages and during interpretation of results. Routinely collected and administrative data are primarily collected for purposes other than research. As a result, not all variables of interest will be available all of the time. For example, within the EXTEND45 Study, there is no information on blood pressure, an important clinical variable in both CKD and diabetes. Depending on the specific research question, surrogates for some of these missing variables may be deployed. For instance, for high blood pressure, a pharmaceutical dispensing of antihypertensive medication or hospital admission for hypertension-related problems may be used. A second limitation is that CKD is primarily defined using laboratory tests. As a result, disease identification is limited to individuals who have presented for medical review and had laboratory tests performed. Conversely, the *absence* of a linked laboratory test in the EXTEND45 Study dataset may be due to (1) the test being performed by a laboratory service provider not part of the EXTEND45 Study, (2) an individual having no indication for a test (and, therefore, healthier), or (3) an individual being indicated but not having access to the appropriate health services. Given the countervailing effects of these different possibilities, a comparison of individuals with linked laboratory data to those without linked laboratory data must be performed in individual analyses. The use of routine laboratory results over time means that inter- and intralaboratory variation may exist. The effects of this are minimized through deep cleaning of the data to identify potentially anomalous results or inconsistent measurement units as well as detailed discussions with laboratory service providers to construct a data dictionary that is specific to their dataset.

Finally, the response rate for the 45 and Up Study baseline questionnaire was 18%, which is similar to other comparable studies. The 45 and Up Study cohort may, therefore, represent a slightly healthier cohort than the general population, with implications for the generalizability of findings that stem from it. Unfortunately, most cohort studies are vulnerable to this healthy volunteer effect. However, we believe that the use of routinely collected data to follow up participants is an excellent alternative to active longitudinal follow-up for minimizing this potential bias over time. Moreover, internal comparisons within the 45 and Up Study cohort have been shown to be generalizable even in the presence of any selection bias [30]. Finally, although not necessarily a limitation, it is important to remember that the different data sources included in the EXTEND45 Study dataset provide varying levels of population coverage; although government datasets typically capture health service use in both the private and public health care sector, the presence of multiple laboratory service providers in NSW means that laboratory data for the participants are not comprehensive over time or across individuals.

### Conclusions

The EXTEND45 Study is, to our knowledge, the first linked data study in Australia to incorporate data from community laboratory service providers with other administrative datasets for chronic disease research. The study represents a unique collaboration among academics, clinicians, primary care researchers, data linkage experts, and laboratory service providers to construct an invaluable data resource that can be used to answer a range of questions relating to chronic disease. Although CKD, diabetes, and CVD have been identified as initial conditions to be examined, consideration of other chronic diseases such as cancer, dyslipidemia, and musculoskeletal conditions is also possible.

## Appendix

Multimedia Appendix 1 Steering committee members of the EXTEND45 Study.

## Abbreviations

AKDN: Alberta Kidney Disease Network
ANZDATA: Australian and New Zealand Dialysis and Transplant
ATC: Anatomical Therapeutic Chemical
CCR: Central Cancer Registry
CHeReL: Center for Health Record Linkage
CKD: chronic kidney disease
CPRD: Clinical Practice Research Datalink
CVD: cardiovascular disease
DHS: Department of Human Services
EXTEND45: EXamining ouTcomEs in chroNic Disease in the 45 and Up Study
HbA_1c_: glycated hemoglobin
HREC: Human Research Ethics Committee
ICD-10-AM: International Statistical Classification of Diseases and Related Health Problems, Tenth Revision, Australian Modification
MBS: Medicare Benefits Schedule
NSW: New South Wales
NSW APDC: New South Wales Admitted Patient Data Collection
PBS: Pharmaceutical Benefits Scheme
PHSREC: Population and Health Services Research Ethics Committee
PPN: project-specific person number
RBDM: Registry of Births, Deaths, and Marriages
RRT: renal replacement therapy
SURE: Secure Unified Research Environment
