# Supplementary material for: EXamining ouTcomEs in chroNic Disease in the 45 and Up Study (the EXTEND45 Study): Protocol for an Australian Linked Cohort Study
Source: JMIR Res Protoc. 2020 Apr 14;9(4):e15646. doi: 10.2196/15646 (PMC7189250; doi:10.2196/15646)
Supplement: Multimedia Appendix 1 [file resprot_v9i4e15646_app1.docx]

**Appendix: Steering Committee Members of the EXTEND45 Study**

| **Role** | **Name and contact details** |
| --- | --- |
| Chair | **A/Prof Meg Jardine**  The George Institute for Global Health  Email: mjardine@georgeinstitute.org.au |
| Study Director | **Dr Carinna Hockham**  The George Institute for Global Health  Email: chockham@georgeinstitute.org.au |
| Director of the Renal & Metabolic Division, The George Institute for Global Health  Content Stream Leader, Tertiary Health Services | **A/Prof Martin Gallagher**  The George Institute for Global Health  Email: mgallagher@georgeinstitute.org.au |
| Content Stream Leader, Chronic Disease | **A/Prof Meg Jardine**  The George Institute for Global Health  Email: mjardine@georgeinstitute.org.au |
| Content Stream Leader, Epidemiology & Methodology | **Dr Min Jun**  The George Institute for Global Health  Email: mjun@georgeinstitute.org.au |
| Content Stream Leader, Health Economics | **Prof Stephen Jan**  The George Institute for Global Health  Email: sjan@georgeinstitute.org.au |
| Content Stream Leader, Primary Health Services | **Prof David Peiris**  The George Institute for Global Health  Email: dpeiris@georgeinstitute.org |
| Content Stream Leader,  Cancer | **A/Prof Germaine Wong**  School of Public Health, University of Sydney  Email: germaine.wong@sydney.edu.au |
| Senior Statistician | **A/Prof Kris Rogers**  The George Institute for Global Health  Email: krogers@georgeinstitute.org |
| 45 & Up Study Representative | The Sax Institute  Email: 45andUp.research@saxinstitute.org.au |
| Content Expertise: Lipidology/Cardiology | **A/Prof David Sullivan**  NHMRC Clinical Trials Centre & Sydney Medical School  Email: david.sullivan@sydney.edu.au |
| Content Expertise: Diabetes | **Prof Sophia Zoungas**  The George Institute for Global Health  Monash Medical Centre  Email: szoungas@georgeinstitute.org.au |
| Content Expertise: Diabetes/Nephrology | **Prof Carol Pollock**  Kolling Institute, Royal North Shore Hospital  Email: carol.pollock@sydney.edu.au |
| Content Expertise: Cardiology/ Health Services | **Prof Clara Chow**  The George Institute for Global Health  Email: cchow@georgeinstitute.org.au |
| Content Expertise: Nephrology/Commercial Delivery of Pharmaceutical Interventions | **Prof John Knight**  The George Institute for Global Health  Email: jknight@georgeinstitute.org.au |
| Content Expertise: Data Linkage Research/ Elderly/Nephrology | **Dr Celine Foote**  The George Institute for Global Health  Email: cfoote@georgeinstitute.org.au |
| Content Expertise: Diabetes/Primary Care | **A/Prof Elizabeth Comino**  Primary and Community Health Research Unit, UNSW  Email: e.comino@unsw.edu.au |
| Content Expertise: Health Service Delivery/Nephrology/Aboriginal Health | **Prof Alan Cass**,  The George Institute for Global Health  Email: alan.cass@menzies.edu.au |
| Sponsor representative (one per sponsor) [non-voting member] | Amgen Pty Ltd.  Merck Sharpe & Dohme Pty Ltd.  Eli Lilly Pty Ltd. |
